# Objectively-measured physical activity patterns and longitudinal weight category status in a rural setting

**DOI:** 10.1186/s13104-019-4660-y

**Published:** 2019-09-23

**Authors:** Ian Cook

**Affiliations:** Physical Activity Epidemiology Laboratory, University of Limpopo (Turfloop Campus), P.O. Box 459, Fauna Park, Polokwane, Limpopo Province 0787 South Africa

**Keywords:** Body composition, Anthropometry accelerometer, Movement monitor, Measurement

## Abstract

**Objectives:**

To investigate the relationship between longitudinal weight-change and objectively-measured physical activity (PA) in a rural African setting in 143 adults (≥ 30 years), using data from two cross-sectional surveys, separated by approximately 10 years. Participants were categorised into three weight-change groups (Weight-loss: ≥ 25 kg m^−2^→ < 25 kg m^−2^; Weight-gain: < 25 kg m^−2^→ ≥ 25 kg m^−2^; Weight-stability: remained < 25 kg m^−2^ or ≥ 25 kg m^−2^). Daily ambulation and activity energy expenditure (AEE), measured in the 2005–2007 health survey, was examined across the weight-change groups. Using the daily AEE data, the proportion of those in the weight-change groups, meeting or not meeting two PA guidelines (150- and 420 min week^−1^), was examined.

**Results:**

Weight-change was found in 18.2% of the sample. There was no significant overall body mass change (+ 1.2 kg, *p *= 0.1616). However, there was significant change in body mass in the weight-gain (+ 15.2 kg) and weight-loss (− 10.8 kg) groups (*p *≤ 0.0011). Nearly 90% of those who gained weight met the 150 min week^−1^ guideline. A significantly greater proportion of the weight-stable group (< 25 kg m^−2^) met the 420 min week^−1^ guideline (*p *< 0.05). Ambulatory level was high irrespective of weight group, although the weight-stable group (< 25 kg m^−2^) approached 15,000 steps day^−1^. There was an inconsistent and weak association between PA and weight-change in this group.

## Introduction

The inclusion of objective measures of physical activity (PA) in population surveillance and longitudinal studies, is now a ubiquitous feature of Physical Activity Epidemiology literature, particularly from industrialized settings [1, 2]. Within the South African setting, particularly rural settings, longitudinal studies which include PA measures are relatively recent developments, and have generally addressed body composition and metabolic variables [3–9]. Not surprisingly, there is a paucity of longitudinal analyses, especially in rural African settings, which have used objective measures of PA [9]. Interestingly, the causal role of PA in weight-change has been challenged, suggesting the importance of other environmental factors [9, 10]. Indeed, sugar-sweetened beverage intake, but not PA, is significantly related to weight gain in low-income, urban and rural South African settings [8]. Similarly, body mass index is associated with socio-economic status, but not PA, in a rural African sample [11], and a recent longitudinal analysis found significant direct effects of socio-economic status on weight-change [3]. Moreover, longitudinal analyses suggest that meeting public health PA guidelines [12] does not translate into an improved weight status [4, 13]. Therefore, the objective of this study was to relate an objective measure of PA to longitudinal weight-change and stability collected in a rural African setting during two cross-sectional surveys, separated by approximately 10 years [14, 15]. More specifically, the analysis probes whether those participants meeting public health PA guidelines are more likely to present with an attenuation of weight gain through weight loss or stability.

## Main text

### Methods

All adults who had sex, age and body mass index (BMI) data across two surveys conducted in the Dikgale Health and Demographic Surveillance System site (DHDSS) [16] in 1997 [14] and 2005–2007 [15], were included in the analysis (male: n = 15; female: n = 128, ≥ 30 years in 1997). Only the 2005–2007 survey included an objective measure of PA. The methodology behind these cross-sectional survey data is described in detail elsewhere [14, 15].

Using body mass (kg) and stature (m), BMI (kg m^−2^) was calculated and classified; under-weight (UW, < 18.5 kg m^−2^), normal weight (NW, 18.5–24.9 kg m^−2^), over-weight (OW, 25–29.9 kg m^−2^), obese (OB, 30–34.9 kg m^−2^) and severely obese (≥ 35 kg m^−2^) [17]. In addition, three weight-change categories were constructed based on BMI changes over the approximately 10-year period (1997 to 2005–2007); weight-loss, -gain or -stability. Due to sample size constraints, BMI change categories were grouped. UW (N = 8) did not differ significantly from NW (N = 37) for age or average steps day^−1^ (*p *> 0.9) and were collapsed into one group. The weight-change categories were defined as follows:

- Weight-loss: OW/OB → UW/NW (≥ 25 kg m^−2^ → < 25 kg m^−2^).
- Weight-gain: UW/NW → OW/OB (< 25 kg m^−2^ → ≥ 25 kg m^−2^).
- Weight stability: UW/NW → UW/NW (< 25 kg m^−2^) and OW/OB → OW/OB (≥ 25 kg m^−2^).

With regard to the 2005–2007 survey data, 7-day accelerometry-based pedometry data were collected using electronic pedometers (NL-2000, New Lifestyles Inc., Kansas City, MO, USA) [15]. Step-based PA public health indices were defined as: sedentary: < 5000 steps day^−1^, low-somewhat active: 5000–9999 steps day^−1^, active: 10,000–12,499 steps day^−1^, very active: ≥ 12,500 steps day^−1^ [18]. A pedometry-based approach was used to estimate the degree to which participants met energy expenditure-based PA public health guidelines [19]. Using daily (kcal kg^−1^ day^−1^) and total weekly AEE (kcal kg^−1^ week^−1^) the following categories were determined:

- ≥ 7.5 kcal kg^−1^ week^−1^, ≥ 1.5 kcal kg^−1^ day^−1^ for ≥ 5 days week^−1^.
- ≥ 21 kcal kg^−1^ week^−1^, ≥ 3 kcal kg^−1^ day^−1^ for 7 days week^−1^.

For the purposes of this analysis a 150- and 420 min week^−1^ standard were used, which equates to ≥ 7.5 kcal kg^−1^ week^−1^ and ≥ 21 kcal kg^−1^ week^−1^, respectively [12, 20].

Descriptive statistics comprised means (one standard deviation) and proportions.

Relationships between categorical variables and differences across multiple group proportions were examined through Fisher’s exact test and *z* tests with correction for multiple comparisons (Bonferroni).

For continuous data, independent and one sample *t* tests examined differences between the sexes and combined data, respectively. One-way Analysis of Variance examined differences across weight-change categories, with post hoc multiple comparison analyses (Sidak’s *t* test) assessing group differences.

To examine average daily step totals across weight-change categories, a Univariate General Linear Model was constructed, adjusting for 2005–2007 survey age. Post hoc multiple comparison analyses (Sidak’s *t* test) assessed group differences.

Two linear regression models were examined for BMI delta (BMI 2005–2007 survey minus BMI 1997 survey, kg m^−2^)—Model 1: age, sex and average daily steps; Model 2: age, sex and average daily AEE. Age and PA variables were obtained from the 2005–2007 survey.

Data were analysed using appropriate statistical software (IBM SPSS Statistics: Release 25 IBM Corporation, Armonk NY, 2017 and GraphPad Prism: version 8.12, GraphPad Software, La Jolla CA, 2019). Significance for all inferential statistics was set at *p *< 0.05.

### Results

There were significant sex-differences in BMI (*p *≤ 0.0014), but not age (*p *≥ 0.0783) for both surveys (Table 1). Proportionally, significantly fewer females were classified as UW/NW (2005–2007 survey, *p *< 0.05), and significantly fewer females showed weight stability in the UW/NW weight-change category (*p *< 0.05) (Table 1). Age distribution (2005–2007 survey) was significantly associated with sex (*p *= 0.0319), and there were significantly more males distributed in the 65+ age group (*p *< 0.05) (Table 1). There were no significant associations between sex and BMI distribution, BMI change distribution, average daily step distribution, and meeting or not meeting PA guidelines (*p *≥ 0.0764). Weight-change was found in 18.2% of the sample while 81.8% maintained their weight status. Given the large proportion of participants who maintained their weight status, this likely explains the similar mean BMI across surveys (Table 1). In addition, the mean body mass change in the weight gain and weight loss groups was + 15.2 kg and − 10.8 kg, respectively (significant difference between groups and change between survey periods, *p *≤ 0.0011). There was no significant difference in body mass change between the weight-stable groups or for the change between survey periods (UW/NW: − 0.8 kg, OW/OB: + 1.3 kg, *p *≥ 0.2320). The overall mean body mass change between the two survey periods was not significant (+ 1.2 kg, *p *= 0.1616).

**Table 1:** Descriptive statistics of male and female participants over two survey periods

	Male (n = 15)	Female (n = 128)	All (n = 143)	*p*-value^‡^
Age (years)^a^				
1997 survey	57.1 (16.4)	50.6 (13.0)	51.3 (13.5)	0.0783
2005–2007 survey	65.9 (16.4)	59.5 (13.1)	60.2 (13.6)	0.0828
Age distribution (2005–2007 survey) (years)				
35–44	20.0 (3)	14.8 (19)	15.4 (22)	0.0319
45–54	6.7 (1)	24.2 (31)	22.4 (32)	
55–64	6.7 (1)	28.1 (36)	25.9 (37)	
65+	66.7 (10)	32.8 (42)^†^	36.4 (52)	
Body mass change (kg)^a^	+ 0.9 (4.1)	+ 1.2 (10.3)	+ 1.2 (9.9)	0.9013
BMI (kg m^−2^)^a^				
1997 survey	23.7 (4.1)	28.1 (6.3)^†^	27.6 (6.3)	0.0014
2005–2007 survey	23.5 (4.2)	28.3 (6.5)^†^	27.8 (6.5)	0.0006
BMI distribution				
1997 survey				
Normal/underweight (< 25 kg m^−2^)	60.0 (9)	39.8 (51)	42.0 (60)	0.1723
Overweight (25–29.9 kg m^−2^)	33.3 (5)	25.0 (32)	25.9 (37)	
Obese (30–34.9 kg m^−2^)	6.7 (1)	19.5 (25)	18.2 (26)	
Severe obesity (≥ 35 kg m^−2^)	0.0 (0)	15.6 (20)	14.0 (20)	
2005–2007 survey				
Normal/underweight (< 25 kg m^−2^)	66.7 (10)	35.9 (46)^†^	39.2 (56)	0.0686
Overweight (25–29.9 kg m^−2^)	26.7 (4)	25.8 (33)	25.9 (37)	
Obese (30–34.9 kg m^−2^)	6.7 (1)	21.1 (27)	19.6 (28)	
Severe obesity (≥ 35 kg m^−2^)	0.0 (0)	17.2 (22)	15.4 (22)	
BMI change distribution (1997 to 2005–2007 survey)				
OW/OB → UW/NW	6.7 (1)	7.8 (10)	7.7 (11)	0.0764
UW/NW → OW/OB	0.0 (0)	11.7 (15)	10.5 (15)	
OW/OB no change	33.3 (5)	52.3 (67)	50.3 (72)	
UW/NW no change	60.0 (9)	28.1 (36)^†^	31.5 (45)	
Ambulation (2005–2007 survey)^a^				
Average steps per day	10,753 (5411)	10,013 (4343)	10,091 (4451)	0.5442
Step distribution (2005–2007 survey)				
Sedentary (< 5000 steps day^−1^)	13.3 (2)	13.3 (17)	13.3 (19)	0.6272
Low-somewhat active (5000–9999 steps day^−1^)	26.7 (4)	41.5 (53)	39.9 (57)	
Active (10,000–12,499 steps day^−1^)	20.0 (3)	18.8 (24)	18.9 (27)	
Highly active (≥ 12,500 steps day^−1^)	40.0 (6)	26.6 (34)	28.0 (40)	
Meeting physical activity guidelines (2005–2007 survey)				
No guidelines	6.7 (1)	13.3 (17)	12.6 (18)	0.6041
150 min week^−1^	60.0 (9)	64.8 (83)	64.3 (92)	
420 min week^−1^	33.3 (5)	21.9 (28)	23.1 (33)	

Raw values reported as %(n) except ^a^mean(sd); ^†^significant difference: male vs. female *p *< 0.05; ^‡^continuous variables: independent *t* test; categorical variables: Fisher’s exact test; UW/NW: (< 25 kg m^−2^), OW/OB: (≥ 25 kg m^−2^)

*BMI* body mass index

**Fig. 1:**
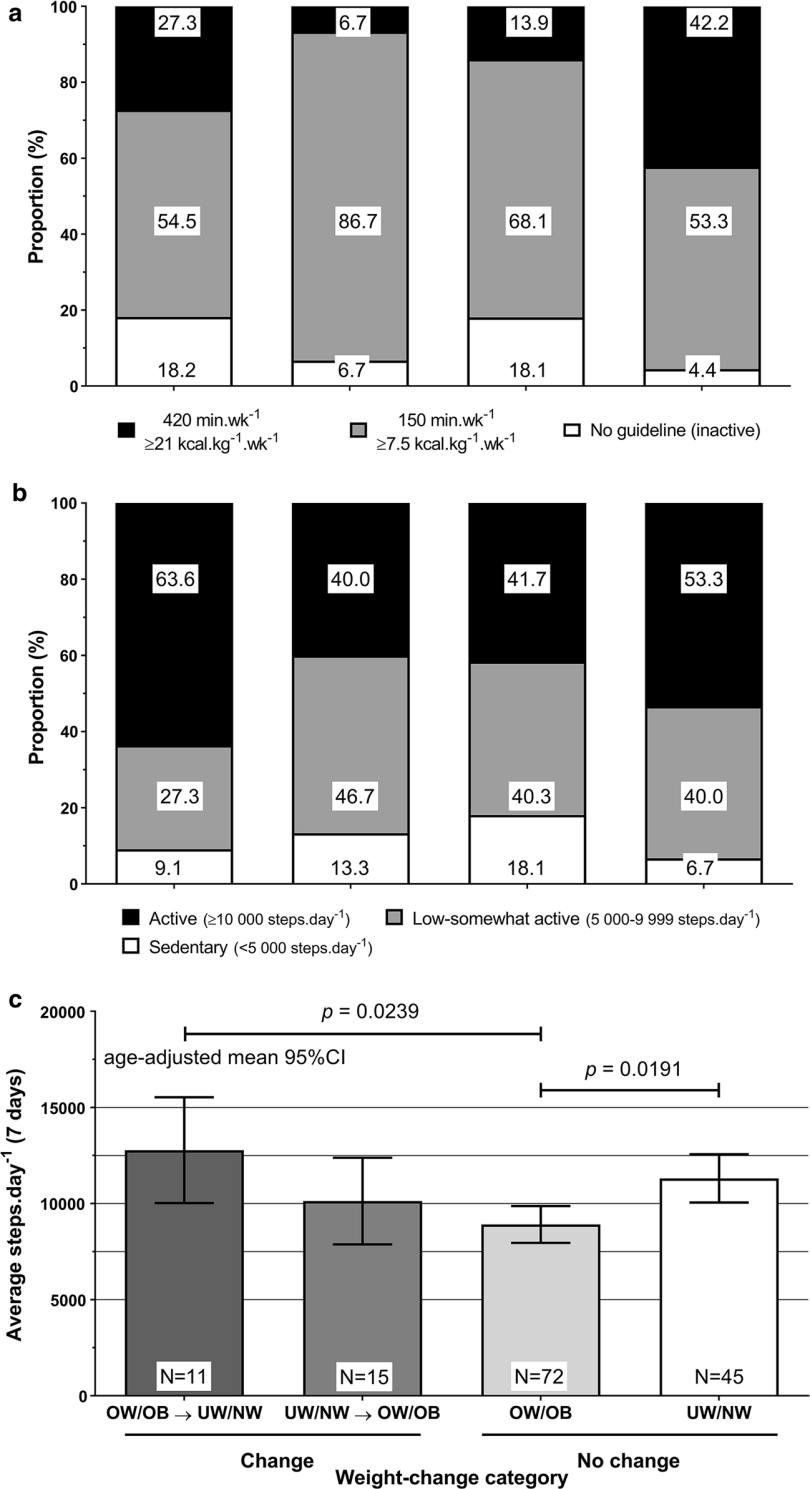
Pedometry-derived measures across weight-change categories. **a** The prevalence of meeting/not meeting energy expenditure, pedometry-based public health guidelines across weight-change categories; **b** the prevalence of step-based public health physical activity guidelines across weight-change categories; **c** the level of average, daily ambulation across weight-change categories. UW = underweight, BMI < 18.5 kg m^−2^, NW = normal weight, BMI = 18.5–24.9 kg m^−2^, OW = overweight, BMI = 25.0–29.9 kg m^−2^, OB = obese, BMI ≥ 30 kg m^−2^

Compared to other weight-change groups, significantly more weight-stable UW/NW achieved the 420 min week^−1^ guideline (*p *< 0.05, Fig. 1a), despite the mean age of 65.2 years. There was no significant association between weight-change category and step-based PA guidelines (*p *= 0.5466, Fig. 1b). The weight-stable UW/NW group were significantly older than the weight-stable OW/OB group (65.2 years vs. 58.3 years, respectively, *p *= 0.0413). Hence, ambulation levels across weight-change category were adjusted for age (Fig. 1c). Weight loss (OW/OB → UW/NW) was associated with significantly higher ambulation level than remaining OW/OB (*p *= 0.0239), approaching levels of up to 15,000 steps day^−1^ (Fig. 1c). There was no significant difference between the change categories (OW/OB → UW/NW: 12,776 steps day^−1^; UW/NW → OW/OB: 10,130 steps day^−1^). Daily average ambulation was significantly higher in weight-stable UW/NW group compared to the weight-stable OW/OB group (11,307 steps day^−1^ vs 8912 steps day^−1^, respectively, *p *= 0.0191) (Fig. 1c).

**Fig. 2:**
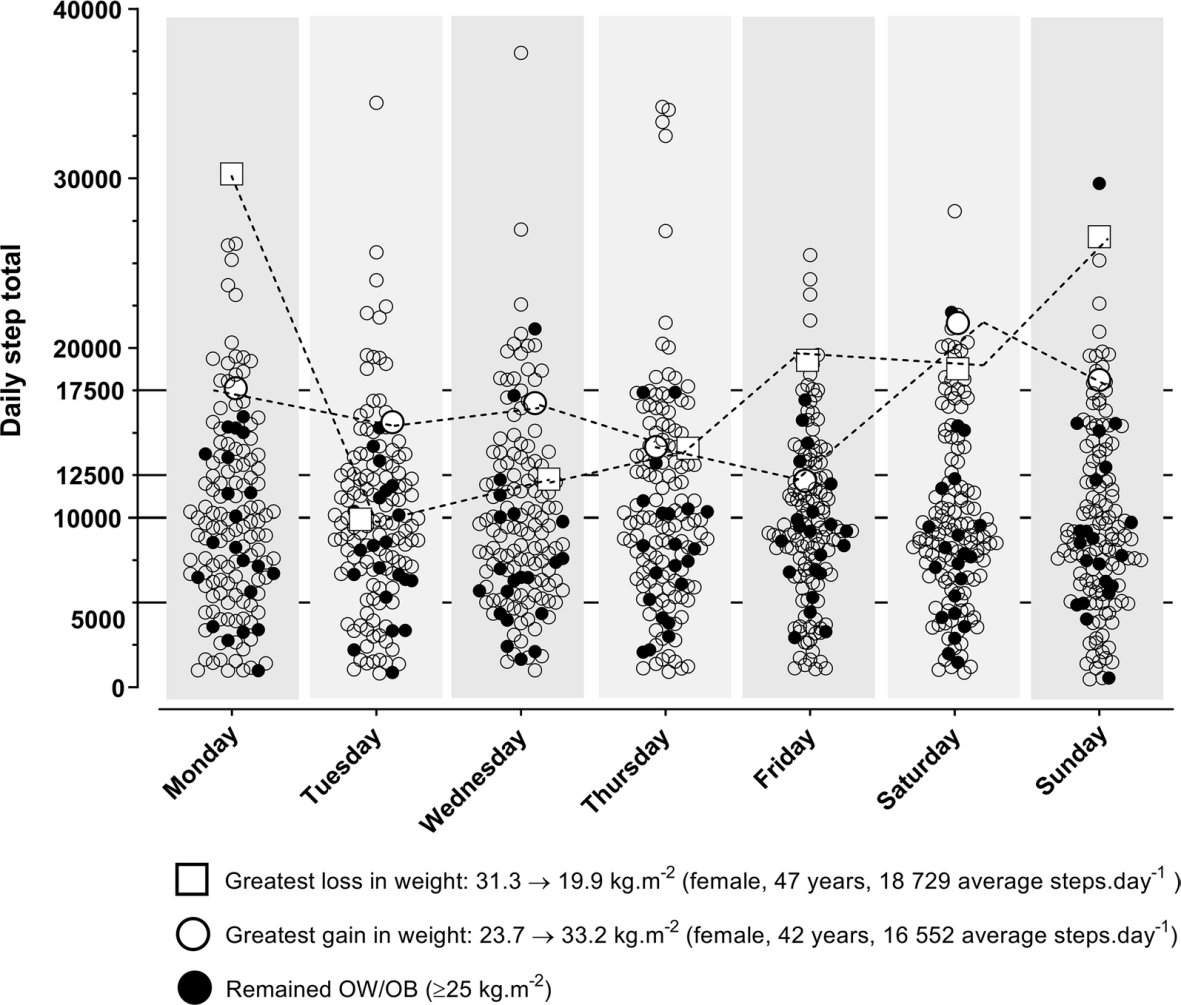
Individual step data across 7 days. Scatterplot of individual, daily ambulation over a full week. Data for a weight-stable group (OW/OB) and two extreme weight-change individuals is highlighted

There was substantial individual variation in daily ambulation levels with values ranging from < 5000 steps day^−1^ to over 20,000 steps day^−1^ (Fig. 2). In contrast, there was surprising homogeneity in daily ambulation levels across two levels of extreme weight gain and weight loss; both these cases averaged above 15,000 steps day^−1^. Of note the high ambulation levels achieved in those who remained OW/OB, with one female achieving 29,697 steps day^−1^ (age = 47.6 years; 2005–2007 survey BMI = 36.3 kg m^−2^) (Fig. 2).

Neither linear regression models for predicting BMI change were significant (Model 1: R^2^ = 0.0299, *p *= 0.2368; Model 2: R^2^ = 0.0526, *p *= 0.0568). For Model 1, none of the predictors were significant (*p *> 0.06). Only average daily AEE was significant in Model 2 (β coefficient = − 0.3600, *p *= 0.0253).

### Discussion

This analysis is novel in that, as far as the author is aware, this is the first longitudinal analysis of weight-change in association with an objective measure of PA, from a rural South African setting.

The main finding of this analysis was that there was no consistent, significant pattern of high sedentary and physical inactivity prevalence in those who remained overweight-obese or moved from underweight-normal weight to obese, and high PA levels in those who remained normal weight or lost weight. In fact, irrespective of the weight-change status, ambulatory PA was high. However, there was a tendency for the weight loss group (OW/OB → UW/NW) and the UW/NW weight-stable group to accrue higher average daily ambulation within the 420 min week^−1^ and ≥ 10,000 steps day^−1^ PA guidelines, with a low proportion in the 150 min week^−1^ guideline. The low sample size might have obscured more definitive and significant patterns. Moreover, although significantly more females were included in the analysis compared with those not included (*p *≤ 0.0006), the mean age, BMI, education status and ambulation were not significantly different (*p *≥ 0.1179) in those who were used for analysis compared to those not, providing some mitigating evidence for the low sample size and convenience sampling.

Interestingly, very similar proportions of those not meeting any energy expenditure-based PA guidelines were seen between quite disparate groups (OW/OB → UW/NW and remained OW/OB: ≈ 18%; UW/NW → OW/OB and remained UW/NW: ≈ 5%). Moreover, more than 80% of any weight-change group adhered to a PA guideline, whether energy expenditure-based or step-based. Previous cross-sectional analyses of the 2005–2007 survey data, have shown that irrespective of increased BMI levels, the ambulation levels and the prevalence of meeting PA guidelines are high [15, 19, 21]. Adult DHDSS residents are active because of daily subsistence and active travel demands, rarely because of sport and recreation, especially amongst females [15, 19].

Meeting PA guidelines, especially 150 min week^−1^ (5 days week^−1^, moderate-to-vigorous intensity), was not associated with weight loss or being weight-stable. Nearly 90% of those who gained weight met the 150 min week^−1^ guideline. These findings are in agreement with Dickie et al. [4] who found that in a group of 57 urban African women, body mass increased over a period of 5.5 years, whether classified as physically active (150 min week^−1^) or physically inactive using a self-report measure. The overall body mass increase was + 7.3 kg [4], which is sixfold higher than the 10 year body mass change in the current rural African sample. However, those meeting PA guidelines were metabolically healthier than those classified as physically inactive [4].

Similarly, in a prospective cohort study (mean follow-up 13.1 years), Lee et al. [13] showed that weight gain was the same in those meeting or not meeting PA guidelines (150 min week^−1^). The overall mean weight gain was 2.6 kg, which is more than two-fold compared to the current sample. Weight stability was evident only in women attaining 420 min week^−1^ of moderate-to-vigorous PA [13]. In the current analysis, only the weight-stable UW/NW group showed a significantly greater prevalence of meeting 420 min week^−1^ PA guidelines (*p *< 0.05). The PA guideline of 420 min week^−1^ [20] addresses issues around weight loss and prevention of weight gain after weight loss [22], unlike the PA guideline of 150 min week^−1^ [12] which addresses risk reduction for mortality and morbidity, and metabolic health [4, 5, 23].

In contrast, an increase in BMI over a 10 year period in 430 urban African women, was significantly, inversely (*p *= 0.02) related to vigorous PA (assessed using a self-report measure). The overall increase in body mass was 5.17 kg [6]. In a more recent analysis, this group has shown the relationship between moderate-to-vigorous PA (150 min week^−1^) and changes in BMI to be part of a complex interaction, with significant direct and indirect effects via socio-economic status. Change in moderate-to-vigorous PA was directly and inversely related to socio-economic status [3].

The generally high levels of PA coupled with high levels of obesity highlighted in the current analysis, are in agreement with the assertion that higher levels of PA do not necessarily attenuate weight gain [9]. In a 2-year prospective cohort (1943 adults of African origin), which included 8-day accelerometry, neither meeting PA guidelines (150 min week^−1^) nor sedentary time were associated with weight gain, suggesting the likelihood that nutritional factors might be of greater importance [9, 10].

In conclusion, this report presents longitudinal weight-change data, incorporating an objective measure of PA, from a rural African setting, which suggests that meeting public health PA guidelines is not tightly associated with weight-change or stability.

## Limitations

Due to the small sample size and cross-sectional, convenience sampling in this study, the results cannot be readily generalized to the rural populations from whence the participants were recruited, nor can causality be shown.

## Data Availability

The data analysed during the current study are not publicly available due to the original consent and ethics approval not containing approval from the participants for data sharing. Reasonable requests would be considered in consultation with the University of Limpopo Ethics Committee and the various community leaders.

## Abbreviations

AEE: activity energy expenditure
BMI: body mass index
DHDSS: Dikgale Health and Demographic Surveillance System Site
NW: normal weight
OB: obese
OW: over-weight
PA: physical activity
UW: under-weight
